# A Case of Double Gallbladder with Adenocarcinoma Arising from the Left Hepatic Duct: A Case Report and Review of the Literature

**DOI:** 10.1155/2010/721946

**Published:** 2010-07-12

**Authors:** Masahiro Kawanishi, Yukio Kuwada, Yutaka Mitsuoka, Shogo Sasao, Teruo Mouri, Eiichi Takesaki, Tadateru Takahashi, Kazuhiro Toyota, Tamaki Nakatani

**Affiliations:** ^1^Department of Gastroenterology, National Hospital Organization, Higashihiroshima Medical Center, Higashihiroshima, 739-0041, Japan; ^2^Department of Surgery, National Hospital Organization, Higashihiroshima Medical Center, Higashihiroshima, 739-0041, Japan

## Abstract

Double gallbladder is a rare congenital biliary anomaly, but an accessory gallbladder arising from the left hepatic duct is a more remarkably rare congenital anomaly. We report a case of double gallbladder with adenocarcinoma and gallstones, which was preoperatively diagnosed by endoscopic retrograde cholangiopancreatography (ERCP) and then confirmed by open laparotomy. A review of the literature is presented.

## 1. Introduction

Double gallbladder is a very rare congenital biliary anomaly with a reported incidence of one per 4,000~5,000 persons [1]. We report one rare case of double gallbladder in which the cystic duct arises from the left hepatic duct. To our knowledge four cases of double gallbladder have been reported in the world in which the double gallbladder arises from the left hepatic duct, but there has been no report of a case in which the accessory gallbladder was complicated with not only gallstones but also adenocarcinoma.

## 2. Case Report

The patient is a 75-year-old male who with chief complaints of repeated epigastralgia and body weight loss from about one month earlier visited our hospital. In November 2009, he was admitted to our hospital for detailed examination and treatment. At time of his admission, there was no evidence of anemia and jaundice, and his abdomen was flat without any tenderness. Laboratory data were normal.

**Figure 1:**
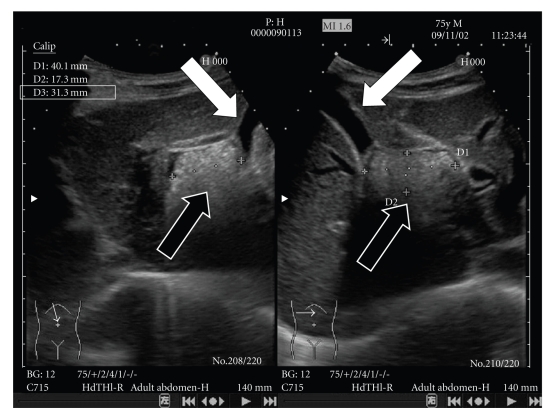
Main gallbladder (white arrow) and cystic structure with multiple stones and debris (black arrow) were confirmed by ultrasonography.

Ultrasonography and computed tomography (CT) of the abdominal region revealed that the main gallbladder was normal without any stones. A cystic structure (40.l mm × 31.3 mm × 17.3 mm) was observed in the upper region of the main gallbladder. It was filled with stones and debris (Figure 1).

**Figure 2:**
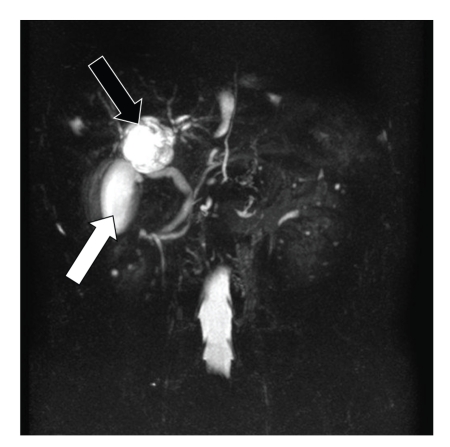
Normal gallbladder (white arrow) and cystic structure (black arrow) were confirmed also by MRCP, but the communication with the biliary tree could not be confirmed.

**Figure 3:**
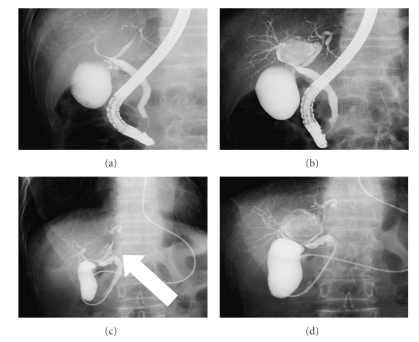
By ERCP the right and left hepatic ducts were displaced (a). With the supplementary injection of radiopaque material, accessory gallbladder could be visualized (b). Cholangiography through ENBD tube conducted the following day showed cystic duct (white arrow) arising from the left hepatic duct (c). Thereafter, an accessory gallbladder was visualized (d).

Magnetic resonance cholangiopancreatography (MRCP) showed findings similar to those of ultrasonography and CT, but the communication between the cystic structure and the hepatic duct could not be defined (Figure 2). By endoscopic retrograde cholangiopancreatography (ERCP) the right hepatic duct and the left hepatic duct were compressed to the left and right, respectively, and with the supplementary injection of radiopaque material an accessory gallbladder with multiple stones was visualized between the left and right hepatic duct (Figures 3(a) and 3(b)).

**Figure 4:**
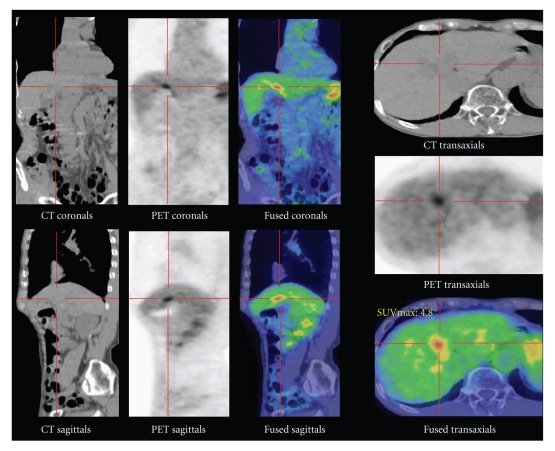
By PET-CT abnormal accumulation of F18-FDP at location considered to be accessory gallbladder could be visualized.

By cholangiography through the endoscopic nazobiliary drainage (ENBD) tube, cystic duct and accessory gallbladder arising from left hepatic duct could be visualized (Figures 3(c) and 3(d)). By positron emission computed tomography (PET-CT) a cystic structure resembling a gallbladder between the left lobe and right lobe of the liver could be visualized together with accumulation of F18-fluroradeoxyglucose (F18-FDP) (Figure 4). The cause of epigastralgia could not be determined by gastroendoscopy and colonoscopy. 

Based on the foregoing findings leading to the possibility of stones and carcinoma in the accessory gallbladder, open cholecystectomy was performed in November 2009. 

**Figure 5:**
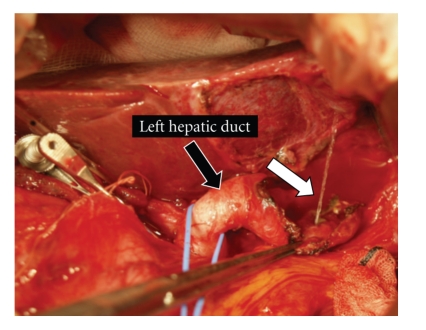
After removing the accessory gallbladder as well as possible, when physiological saline solution was injected into the left hepatic duct, there was a vigorous emission of this solution from the accessory gallbladder (white arrow). After confirming the exit of the cystic duct toward the accessory gallbladder, the exit was ligated.

**Figure 6:**
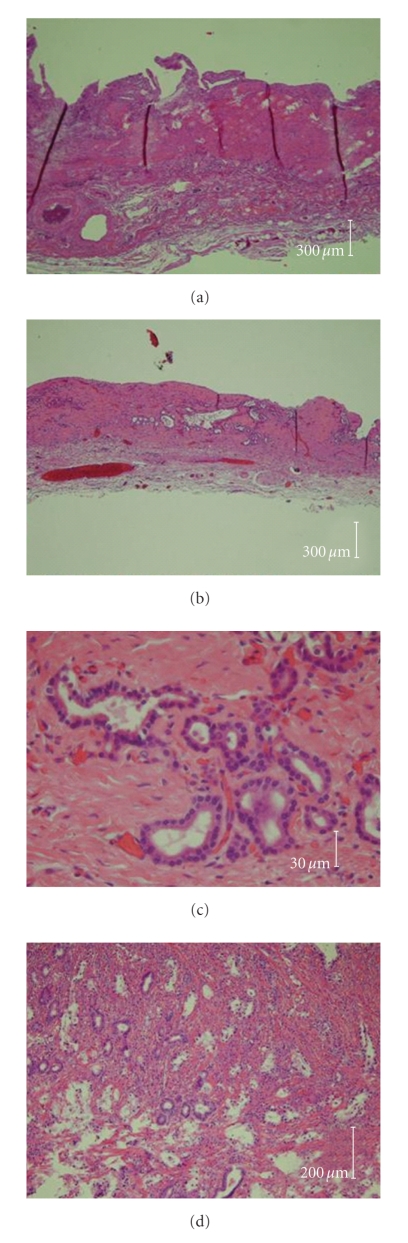
Microscopic image of main gallbladder (a), accessory gallbladders ((b), (c)), and tubular adenocarcinoma in the accessory gallbladder (d). Lobular hyperplasia or adenosis was observed in the accessory gallbladder, but the basic structure of gallbladder (mucosal layer, muscular layer, and submucosal layer) could be seen in both gallbladders. Tubular adenocarcinoma infiltrating into the submucosal layer could be confirmed.

Following the surgical removal of the main gallbladder, one-half of the accessory gallbladder was found to be embedded in the liver. The accessory gallbladder could be separated from the liver, but as the separation of the accessory gallbladder from the left and right hepatic duct was difficult due to severe inflammation, the accessory gallbladder was opened and after excluding the stones therein, the wall of the accessory gallbladder was removed as much as possible. After confirming the exit of the cystic duct toward the accessory gallbladder, ligation was made (Figure 5). The remaining gallbladder wall was coagulated by argon beam. Well differentiated tubular adenocarcinoma was observed in the resected accessory gallbladder with infiltration into the subserosal layer (Figure 6).

The patient has been discharged, but is now under postoperative chemotherapy as an outpatient.

## 3. Discussion

**Figure 7:**
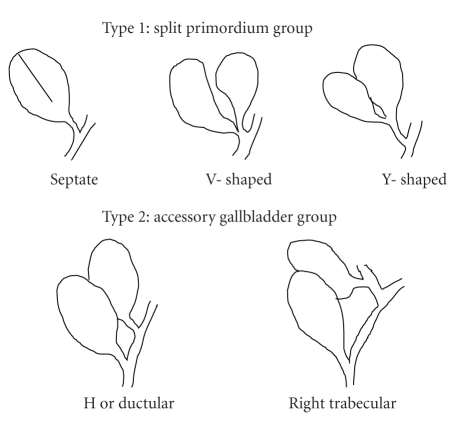
Double gallbladder as classified by Harlaftis et al. [2].

Double gallbladder is a rare congenital anomaly and it has been reported in 2 cases (0.02%) among 9921 autopsy cases and in 3 cases (0.03%) among 9970 cases in a radiographic survey [1]. Boyden has classified double gallbladder into two types; Y type in which two cystic ducts are united to connect with the common bile duct and H type in which two cystic ducts are separately united to common bile duct or hepatic duct. Classification by Harlaftis et al. [2] defines based on morphology and embryogenesis two main groups and a third miscellaneous group. The former is characterized by the presence of a single cystic duct entering the common bile duct (Type l, that is split primordium group). The accessory gallbladder group is characterized by two cystic ducts opening separately into the biliary tree (Type 2, that is accessory gallbladder group). The last is Type 3, the miscellaneous group which does not fall into the foregoing two types (Figure 7). 

Singh et al. [4] in their review in 2006 of 148 cases of double gallbladder reported according to the classification of Harlaftis et al. belonging to Type 1 there were 16 cases (10.8%) of septate gallbladder subtype, 14 cases (9.5%) of bilobed or V shaped subtype, and 36 cases (24.3%) of Y shaped subtype and belonging to Type 2, there were 72 cases (48.6%) of H or ductular subtype and 4 cases (2.7%) of trabecular subtype connected to the right hepatic duct, but no case connected to the left hepatic duct. 

**Figure 8:**
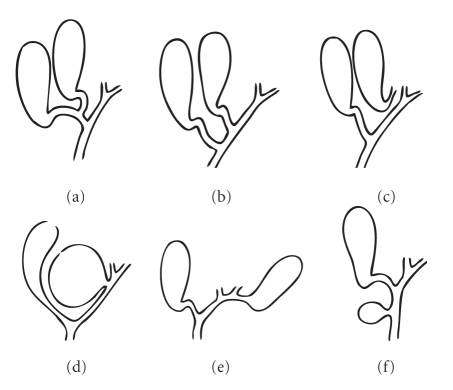
Double gallbladder as classified by Gross [3].

Furthermore, in 1969 Gross [3] in his report on congenital anomalies of the gallbladder reviewed 28 cases of double gallbladder and made a classification by A-F type (Figure 8). He has presented a case connected to the left hepatic duct as Type E. Thereafter, Gorecki et al. [5] reported a case of double gallbladder in a 69-year-old female with stones in both gallbladders with the accessory gallbladder originating from left hepatic duct, but the cystic duct could not be visualized. In 2009, Kim et al. [6] have reported duplicate gallbladder with stones arising from the left hepatic duct in a 78-year-old male 10 years after an open cholecystectomy. An apparent spiral structure of the cystic duct could not be identified in this case.

**Figure 9:**
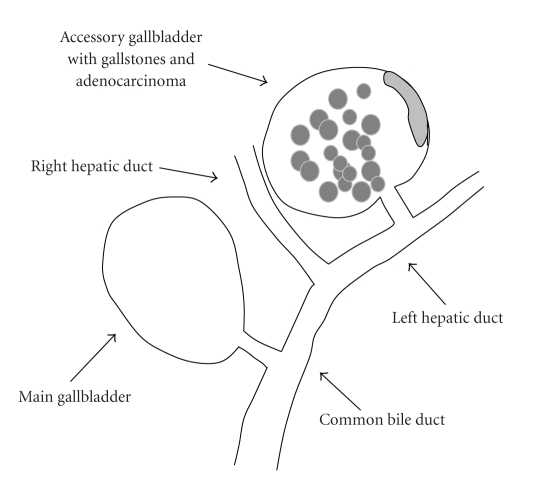
Illustration of our case shows double gallbladder with adenocarcinoma and gallstones arising from the left hepatic duct.

Our case appears to correspond to H type reported by Boyden, type 3 or type 2 (left trabecular subtype) reported by Harlaftis et al., and type E reported by Gross. It is thus the fourth reported case in which the accessory gallbladder arises from the left hepatic duct (Figure 9). The accessory gallbladder not only has stones but also adenocarcinoma. The cystic duct could also be observed by imaging diagnosis.

Abdominal ultrasonography and CT are available as simple imaging modality of double gallbladder and the presence of mass can be identified, but the relationship with the biliary tree remains obscure. By MRCP the location of the mass in relation to the biliary tree can be better identified than by CT and ultrasonography, but it is difficult to determine whether it is united, in fact, with the biliary tree. ERCP is an invasive procedure, but by injection of radiopaque material and by careful observation of the injection course it is possible to visualize the cystic duct and the adjacent accessory gallbladder. For the evaluation of the biliary tree, this is the most valuable diagnostic modality. Also, in our present case, by ERCP followed thereafter by cholangiography through the ENBD tube, the cystic duct connecting to the left hepatic duct and gallbladder with multiple stones could be confirmed. Furthermore, by PET-CT abnormal accumulation of F18-fluroradeoxyglucose was observed, suggesting the presence of carcinoma of the gallbladder. 

Double gallbladder is often discovered accidentally or by complications of gallstones and cholecystitis. The accompanying symptoms are loss of appetite, nausea, vomiting, and epigastralgia. Also, in our case, the chief complaints were loss of appetite and epigastralgia without abnormalities in the laboratory data and gastroendoscopy and colonoscopy did not suggest the underlying cause. From the observed symptoms the complications of gallstones and cholecystitis were considered to be contributory. 

There have been double gallbladder cases who underwent successful laparoscopic cholecystectomy [7, 8], but for type l of the classification of Harlaftis et al. laparoscopic cholecystectomy is also possible. In Type 2, the possibility of injury to the bile duct and hepatic artery is high with the successful cases of laparoscopic cholecystectomy being small in number [9, 10]. In our case, being accessory gallbladder arising from the left hepatic duct with suspected complication of carcinoma, open laparotomy was selected. 

In the pathological findings, adenosis was observed in the accessory gallbladder with normal three layer structure without evident findings of adenomyomatosis. With regard to mechanism of complication of gallstones and carcinoma, due to functional disorder of contraction in the accessory gallbladder, carcinogenesis accompanied by gallstones and inflammation is speculated, but the details involved remain unknown.

As adequate confirmation was not possible in our case by CT and MRCP, ERCP and cholangiography through ENBD tube enabled diagnosis of accessory gallbladder with adenocarcinoma arising from left hepatic duct. 

Though double gallbladder is an extremely rare congenital anomaly, the biliary tree could be accurately confirmed by ERCP, permitting surgery without any intraoperative injury.
